# Genome-wide association study identifies SNPs for growth performance and serum indicators in Valgus-varus deformity broilers (*Gallus gallus*) using ddGBS sequencing

**DOI:** 10.1186/s12864-021-08236-3

**Published:** 2022-01-06

**Authors:** Yaping Guo, Hetian Huang, Zhenzhen Zhang, Yanchao Ma, Jianzeng Li, Hehe Tang, Haoxiang Ma, Zhuanjian Li, Wenting Li, Xiaojun Liu, Xiangtao Kang, Ruili Han

**Affiliations:** College of animal science and technology, Henan Agricultural University, Zhengzhou, Henan Province 450002 P.R. China

**Keywords:** Broiler, Valgus-varus deformity, Genome-wide association study, Growth performance, Serum indicators

## Abstract

**Background:**

Valgus-varus deformity (VVD) is a lateral or middle deviation of the tibiotarsus or tarsometatarsus, which is associated with compromised growth, worse bone quality and abnormal changes in serum indicators in broilers. To investigate the genetic basis of VVD, a genome wide association study (GWAS) was performed to identify candidate genes and pathways that are responsible for VVD leg disease, serum indicators and growth performance in broilers.

**Results:**

In total, VVD phenotype, seven serum indicators and three growth traits were measured for 126 VVD broilers (case group) and 122 sound broilers (control group) based on a high throughput genome wide genotyping-by-sequencing (GBS) method. After quality control 233 samples (113 sound broilers and 120 VVD birds) and 256,599 single nucleotide polymorphisms (SNPs) markers were used for further analysis. As a result, a total of 5 SNPs were detected suggestively significantly associated with VVD and 70 candidate genes were identified that included or adjacent to these significant SNPs. In addition, 43 SNPs located on Chr24 (0.22 Mb - 1.79 Mb) were genome-wide significantly associated with serum alkaline phosphatase (ALP) and 38 candidate genes were identified. Functional enrichment analysis showed that these genes are involved in two Gene Ontology (GO) terms related to bone development (cartilage development and cartilage condensation) and two pathways related to skeletal development (Toll−like receptor signaling pathway and p53 signaling pathway). *BARX2* (BARX homeobox 2) and *Panx3* (Pannexin 3) related to skeleton diseases and bone quality were obtained according to functional analysis. According to the integration of GWAS with transcriptome analysis, *HYLS1* (HYLS1 centriolar and ciliogenesis associated) was an important susceptibility gene.

**Conclusions:**

The results provide some reference for understanding the relationship between metabolic mechanism of ALP and pathogenesis of VVD, which will provide a theoretical basis for disease-resistant breeding of chicken leg soundness.

**Supplementary Information:**

The online version contains supplementary material available at 10.1186/s12864-021-08236-3.

## Background

Valgus-varus deformity (VVD), a common leg disorder in broilers and turkeys, is characterized by a lateral (outward) or middle (inward) angulation of the tibiotarsal bone that results in deviation of the tarsometatarsus [1]. It has caused severe economic losses for the broiler industry and physical distress in broilers [2, 3]. Previous study showed that VVD had compromised growth, worse bone quality, calcification disorders and abnormal changes in serum lipid indicators, serum calcium (Ca), phosphorus (P) and serum alkaline phosphatase (ALP) levels [4].

In humans and animals, the blood components are considered to reflect immune activity and nutrient metabolism. Serum Ca, P and serum ALP are important and evaluable indicators for bone metabolism and mineral status [5]. ALP is the by-production during bone formation and biochemical marker of bone metabolism, which can reflect bone development state [6]. Abnormal lipid metabolism is an important factor to leg disorder in broiler [7]. Triglyceride (TG), total cholesterol (T-CHO), high-density lipoprotein cholesterol (HDL-C), and low-density lipoprotein cholesterol (LDL-C) in serum are four representational indicators for lipid metabolism. Bodyweight is an important trait to estimate the profitability of broiler production. Furthermore, strong genetic correlations have been found between body weight and VVD incidence in broiler production [3]. Shank length and girth are two important traits for the development of skeleton in broiler [8, 9]. Therefore, these traits are directly or indirectly associated with VVD in broiler.

Up to now, the precise etiology and pathogenesis of VVD still remain uncertain [3]. The incidence of VVD has been reduced effectively by genetic selection in the UK [10]. The heritability or pseudo heritability of leg disorders range from 0.10 to 0.40 [11, 12]. In our previous study, some candidate genes associated with VVD were found based on case/control design with VVD and normal broilers using RNA sequencing [10]. These data indicated that genetic factors will provide a more scientific and reasonable explanation for pathogenesis of VVD. Nevertheless, the molecular and regulatory mechanisms in VVD are very limited.

Genome-wide association studies (GWAS), a mature analysis way, have been extensively used to identify single nucleotide polymorphisms (SNPs) related to complex diseases or traits during the last decade [13, 14]. To the best of our knowledge, no large-scale GWAS has been performed to identify genomic loci and candidate genes for VVD in broilers. In this study, the objectives were to identify genetic markers and candidate genes that are associated with VVD leg disease and its related traits including seven serum indicators and three growth traits by performing a GWAS. We conducted a GWAS in 248 Hubbard broilers using genotyping-by-sequencing (GBS). The results could provide new insights into the molecular mechanism of VVD and increase the understanding of the relationship between VVD and serum indicators in healthy leg breeding in birds.

## Results

### Phenotype information and SNP characteristics after quality control

The phenotype information for growth performance, serum indicators and leg disorder were listed in Table S1. In this study, a total of 233 birds were retained after quality control of the genotype, including 120 VVD birds (case group) and 113 sound birds (control group). A number of 256,599 SNPs were obtained for further association analysis. All SNPs were uniformly distributed on each chromosome and these SNPs are mostly located in intron (Fig. S1) and intergenic regions (Fig. S2).

### GWAS analysis for VVD trait

**Fig. 1:**
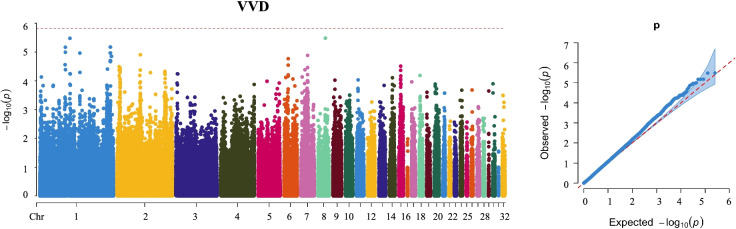
Manhattan and quantile-quantile (QQ) plot on VVD traits at genome-wide level. Manhattan plot is on the left and QQ plot is on the right. The horizontal red lines indicated the whole-genome significant (*P* = 1.51e-6)

**Fig. 2:**
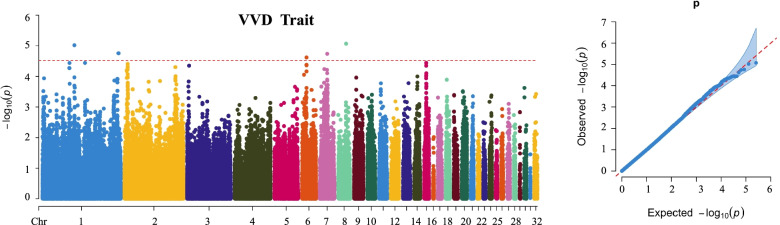
Manhattan and quantile-quantile (QQ) plot on VVD traits at suggestive level. Manhattan plot is on the left and QQ plot is on the right. The horizontal red lines indicated the whole-genome significant (*P* = 3.02e-5)

Pairwise kinship was estimated using autosomal SNP information (Fig. S3), which indicated no population stratification. For VVD, the manhattan and QQ plots indicated that there was no significant SNP associated with VVD clinical phenotype (Fig. 1). At the suggestive significant level, a total of 5 SNP was detected, which were located on Chr 1 (rs80839937, rs192358715), Chr 7(rs18584148), Chr 6 (rs12086380) and Chr 8 (rs20132188), respectively (Fig. 2 and Table 1). The most significant SNP was rs20132188 (*P* = 8.49 × 10 ^− 6^) at Chr 8. A total of 70 putative candidate genes were obtained by annotating each significant locus (Table S2).

**Table 1:** Loci associated with VVD trait

Trait	SNP	Chr	Position	REF	ALT	*P*-value
VVD	rs20132188	8	S8_20132188	C	A	8.49E-06
	rs 80,839,937	1	S1_80,839,937	G	A	9.51E-06
	rs192358715	1	S1_192358715	A	G	1.74E-05
	rs 18,584,148	7	S7_18,584,148	A	G	1.82E-05
	rs 12,086,380	6	S6_12,086,380	C	T	2.39E-05

### GWAS analysis for serum indicators and growth performance

**Fig. 3:**
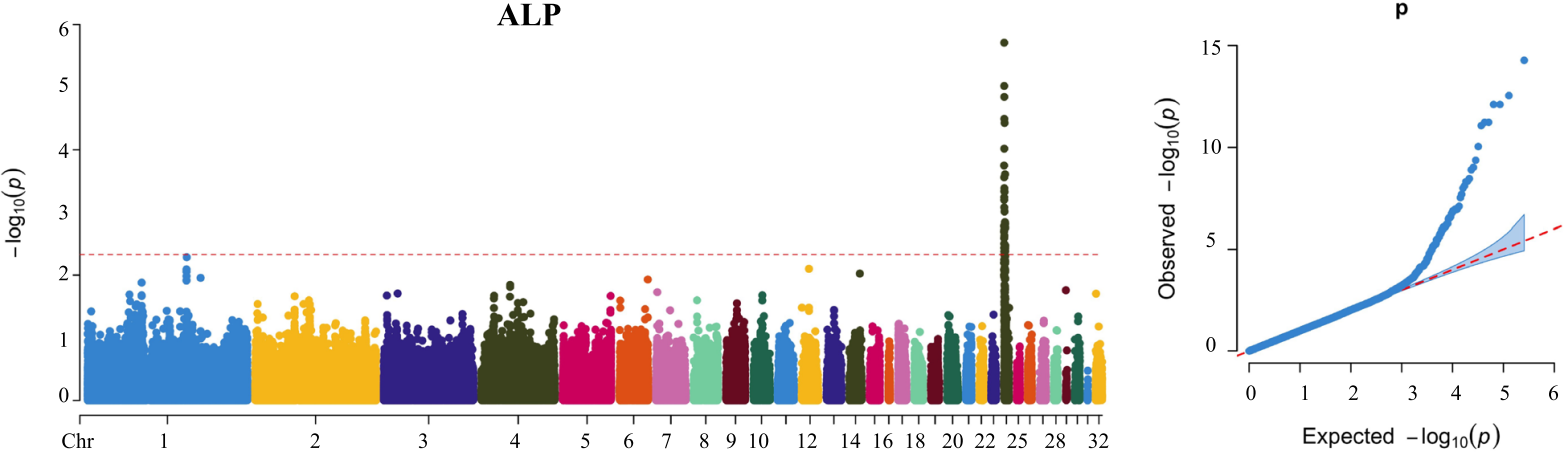
Manhattan and quantile-quantile (QQ) plot revealing the significance signal associated with ALP levels in broiler. Manhattan plot is on the left and QQ plot is on the right. The horizontal red lines indicated the whole-genome significant (*P* = 1.51e-6)

**Fig. 4:**
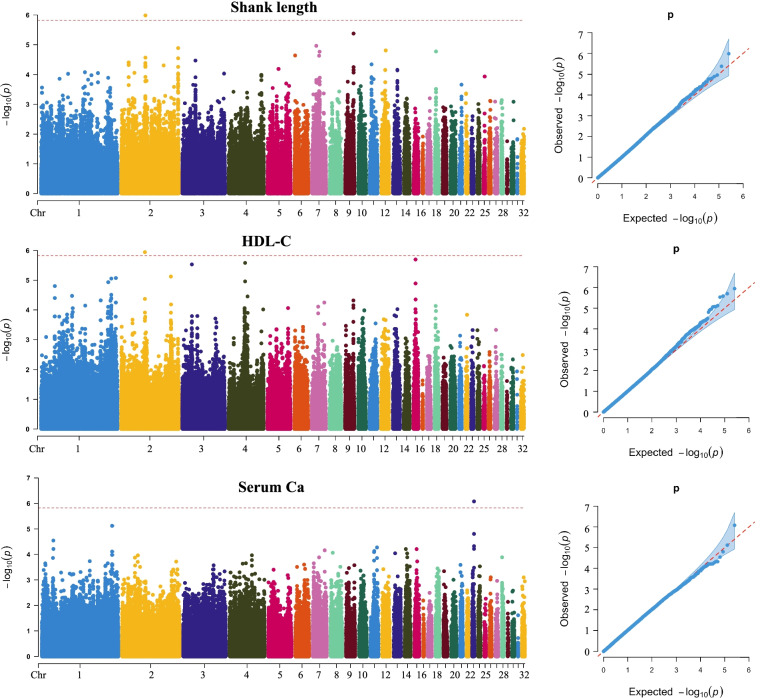
Results of genome-wide association analysis for Shank length, HDL-C and Serum Ca. Manhattan and quantile-quantile (QQ) is on the left and right, respectively

A total of 43 genome-wide significant SNPs were identified for serum ALP, which were located region at 0.22 Mb to 1.79 Mb on Chr 24 (Fig. 3 and Table 2). The most significant SNP was rs249241 (*P* = 5.36 × 10^− 15^, Position: 0.47 Mb). The estimated genomic inflation factor (λ) for ALP was 1.0, which indicated the low false positives to analysis results. For H-HDL, a significant SNP (rs71661, *P* = 1.14 × 10^− 6^) was identified on Chr 2 at position 61.93 Mb (Fig. 4 and Table 3). A Manhattan plot revealed that univariate analysis generated a *P*-value that exceeded the Bonferroni cutoff on Chr 23, which illustrates that SNP was significantly associated with Serum Ca (rs248988, *P* = 8.34 × 10^− 7^, Position: 4.68 Mb) (Fig. 4 and Table 3). The manhattan and QQ plots for TG, T-CHO, LDL-C, serum P were indicated in Fig. S4. Association analysis results revealed that these SNP were not significant by Manhattan plot. A top SNP (rs174201, *P* = 2.06 × 10^− 6^, Position: 24.62 Mb) was focused for TG on Chr 2 (Fig. S4). For T-CHO, the top SNP was rs121566 (*P* = 3.60 × 10^− 6^) on Chr 3 at position 92.15 Mb (Fig. S4). As the top SNP for LDL-C, the rs90406 (*P* = 3.74 × 10^− 6^) was detected which was located on Chr 2 at 132.19 Mb (Fig. S4). For HDL-C level, Fig. S4 showed that a top SNP rs71661 (*P* = 1.14 × 10^− 6^) was located on Chr 2 at 61.3 Mb. The genomic control inflation factor (λ) calculated for serum indicators ranged from 0.99 to 1.01, indicating acceptably low false positives, and revealing relatively good consensus between the observed distributions of the *P*-value and expected.

**Table 2:** Loci associated with ALP

Trait	SNP	Chr	Position	REF	ALT	*P*-value
ALP	rs249241	24	S24_475471	G	A	5.36E-15
	rs249240	24	S24_475460	C	T	2.79E-13
	rs249257	24	S24_566501	C	T	7.65E-13
	rs249258	24	S24_566558	C	A	7.65E-13
	rs249251	24	S24_551994	A	G	5.76E-12
	rs249252	24	S24_552024	A	G	5.76E-12
	rs249338	24	S24_935640	T	C	8.40E-12
	rs249247	24	S24_534252	C	T	9.00E-11
	rs249221	24	S24_310849	G	A	4.26E-10
	rs249495	24	S24_1517358	T	C	9.56E-10
	rs249235	24	S24_432460	G	A	1.27E-09
	rs249267	24	S24_602600	T	A	3.45E-09
	rs249242	24	S24_488344	G	A	4.56E-09
	rs249334	24	S24_928929	A	T	4.82E-09
	rs249232	24	S24_422686	C	T	7.96E-09
	rs249234	24	S24_432376	G	A	1.01E-08
	rs249243	24	S24_488395	A	G	2.02E-08
	rs249317	24	S24_851132	T	C	2.96E-08
	rs249450	24	S24_1377319	C	T	7.82E-08
	rs249544	24	S24_1711218	C	T	8.96E-08
	rs249212	24	S24_229128	C	T	1.11E-07
	rs249213	24	S24_229158	A	G	1.11E-07
	rs249214	24	S24_229193	T	C	1.11E-07
	rs249562	24	S24_1799291	G	A	1.28E-07
	rs249563	24	S24_1799295	A	G	1.28E-07
	rs249233	24	S24_431876	G	A	1.56E-07
	rs249215	24	S24_229513	C	G	1.84E-07
	rs249272	24	S24_622253	T	C	2.61E-07
	rs249263	24	S24_578123	C	T	2.87E-07
	rs249264	24	S24_578225	G	A	2.87E-07
	rs249226	24	S24_363314	C	A	3.33E-07
	rs249225	24	S24_362451	G	A	5.54E-07
	rs249288	24	S24_725392	T	C	5.71E-07
	rs249582	24	S24_1857293	C	T	6.65E-07
	rs249554	24	S24_1735874	C	G	6.71E-07
	rs249223	24	S24_362315	T	C	8.10E-07
	rs249224	24	S24_362316	G	A	8.10E-07
	rs249407	24	S24_1270484	C	A	8.23E-07
	rs249550	24	S24_1735676	C	T	8.26E-07
	rs249547	24	S24_1721382	G	A	1.10E-06
	rs249237	24	S24_472868	G	A	1.13E-06
	rs249289	24	S24_767245	A	G	1.13E-06
	rs249238	24	S24_474682	A	G	1.51E-06

**Table 3:** Loci associated with Shank length, Serum Ca and H-HDL

Trait	SNP	Chr	Position	REF	ALT	*P*-value
Shank length	rs72002	2	S2_63028430	A	G	1.04E-06
H-HDL	rs71661	2	S2_61933707	A	C	1.14E-06
Serum Ca	rs248988	23	S23_4682510	T	C	8.34E-07

The GWAS identified a SNP that was significantly associated with shank length, and the QQ plot for this trait supported the results shown in the Manhattan plot (Fig. 4). The significant SNP (rs72002, *P* = 1.04 × 10^− 6^) was identified on Chr 2 at position 63.02 Mb (Table 3). The manhattan and QQ plots for body weight and shank girth were indicated in Fig. S5. Association analysis results revealed that these SNP were not significant by Manhattan plots. For body weight, Fig. S5 indicated that a top SNP was rs230254 (*P* = 4.02 × 10^− 6^) located on Chr 15 at position 5.79 Mb. A top SNP site (rs199378, *P* = 1.20 × 10^− 5^) for shank girth was detected on Chr 9 at 14.63 Mb (Fig. S5). The genomic control inflation factor (λ) calculated for the growth performance ranged from 0.99 to 1.11.

### Linkage disequilibrium (LD) blocks analysis

**Fig. 5:**
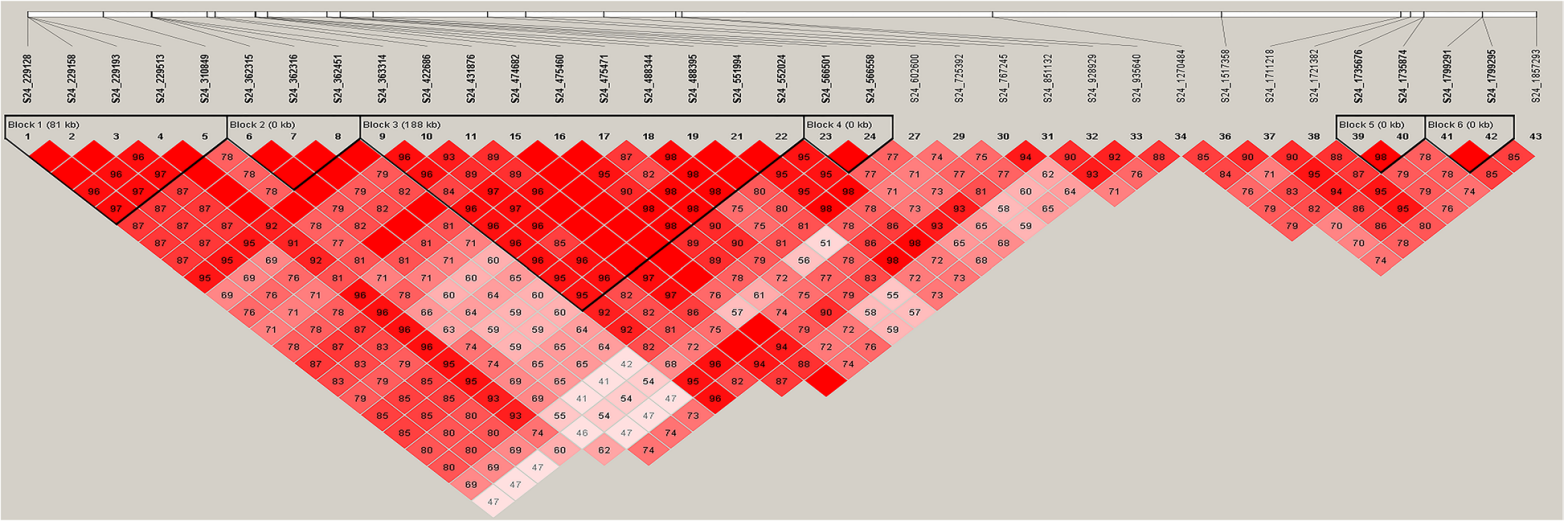
LD blocks with SNPs which affect ALP levels

For Serum ALP, we performed LD blocks analysis of 43 significant SNPs on Chr 24. After haplotype construction with these SNPs, two smaller blocks were obtained at 0.23–0.31 Mb and 0.36–0.55 Mb (Fig. 5). Strong linkage disequilibrium was observed among 5 SNPs, which were located in the other haplotype. In addition, 10 SNPs were located in one haplotype block. The result hinted that 2 regions were important to find the true causal mutations SNP.

### Candidate genes and functional annotation

For VVD trait, 70 candidate genes that included or adjacent to the 5 significant SNPs with a distance less than 300 kb were annotated (Table S3). GO and pathway analysis were performed to determine the biological functions of the 70 protein coding genes. Uniplex complex GO term (GO:1990246) was the most significant GO term in GO enrichment analysis results (Table S4). KEGG enrichment analysis results revealed that Hedgehog signaling pathway (gga04340) and Wnt signaling pathway (gga04310) were important to skeleton development and leg disorder (Table S5).

**Fig. 6:**
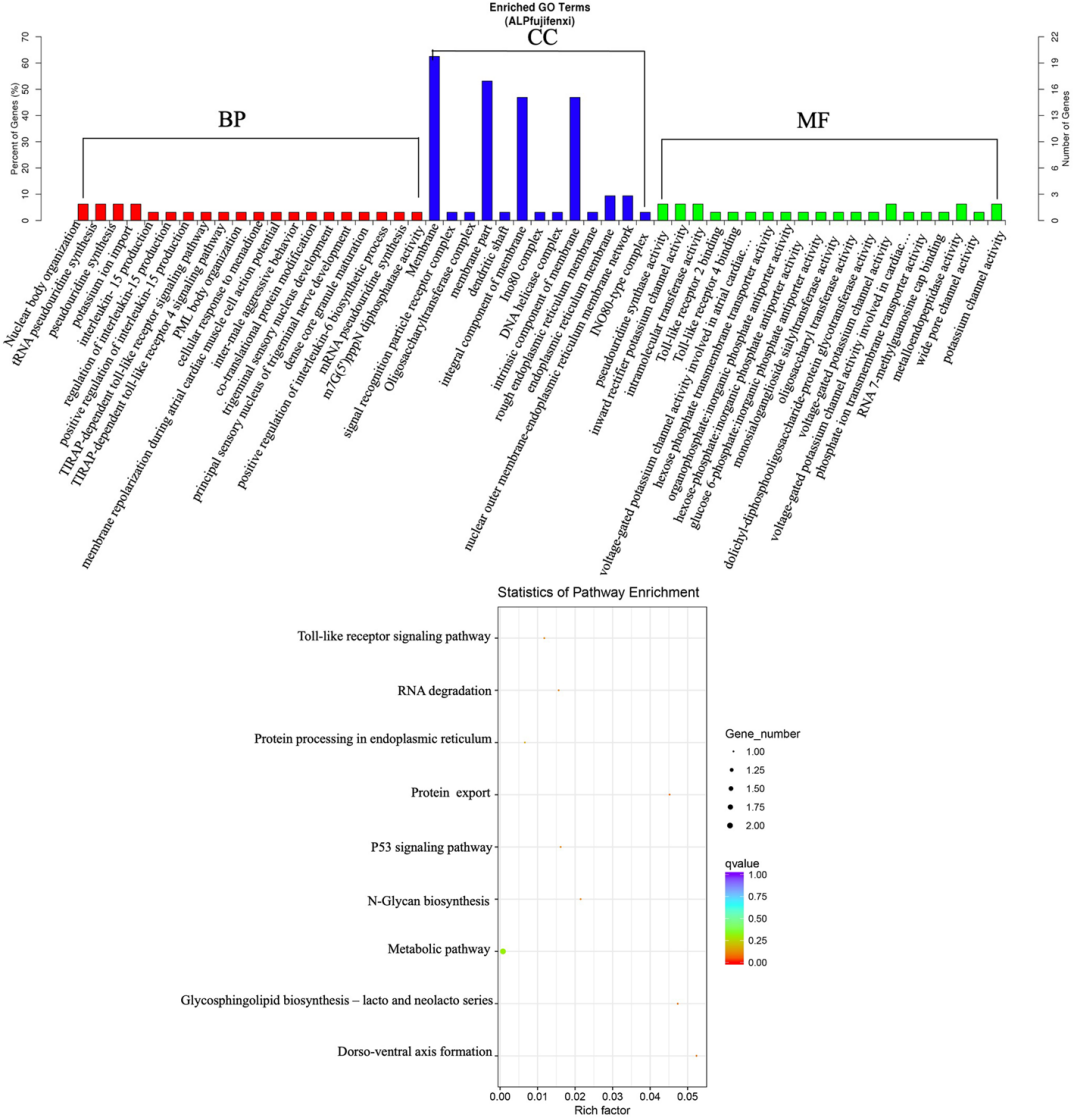
Comparison of functional annotations for the candidate genes with ALP. Histogram representing GO classification results for t the candidate genes on the top. The most enriched KEGG pathways on the underneath

Putative candidate genes of each significant locus were annotated. A total of 38 protein coding genes were obtained for ALP (Table S6). GO and KEGG pathway enrichment analysis were performed on 38 protein coding genes (Fig. 6, Table S6 and Table S7). Among GO enrichment analysis results, nuclear body organization (GO: 0030575) was the most significant GO term in biological process. Within molecular function, pseudouridine synthase activity (GO: 0009982) was the most dominant GO subcategories. Regarding the cellular component category, axon (GO: 0030424) was the most significant GO term. It is worth mentioning that *BARX2* (BARX homeobox 2) was enriched to cartilage development (GO: 0051216) and cartilage condensation (GO: 0001502) GO term. Pathway enriched analysis results showed that Toll−like receptor signaling pathway (gga04620) and p53 signaling pathway (gga04115) were important to bone development and bone disease. By integrating analysis of GWAS and previous transcriptome results, we identified *HYLS1* (centriolar and ciliogenesis associated) as an important susceptibility gene.

## Discussion

A total of 5 SNPs were detected for VVD trait at the suggestive significant level. Among these SNPs, the rs18584148 was located on Chr7. Notably, a study indicated that the regions related to chicken leg disease score on chromosome 7 by the ssGWAS methods [15]. Thus, the findings illustrated that the chromosome 7 merit closer attention to leg disorder in further search. Furthermore, five SNPs were performed relevant gene annotation, which obtained 70 candidate genes. Among these candidate genes, the low-density lipoprotein receptor-related protein (LRP2) plays important role in lipoprotein metabolism progress. Our previous study indicated that an association between VVD and lipid metabolism in broilers [4]. In *LRP2* knockout mice, bone disorder and Vitamin D deficiency were found. In human, *LRP2* is closely related to bone mineral density and bone quality. These data revealed that *LRP2* gene merits closer attention in our future study. A total of 70 candidate genes were performed functional enrichment analysis. Our previous study demonstrated that VVD mainly reduces skeletal quality and development to broilers [4]. Significantly, Hedgehog signaling pathway (gga04340) and Wnt signaling pathway (gga04310) were closed related to skeletal development and bone homeostasis. Therefore, these SNPs, genes and pathways may provide new insights for understanding the relationship between VVD and genetics in further study.

The etiology and pathogenesis of VVD were complex and multiple factors, including genetics, exercise, nutrient distribution, growth rate and lighting program [3]. The heritability or pseudo heritability of leg disorders range from 0.10 to 0.40 [11, 12]. The incidence of VVD has been reduced effectively decreased by genetic selection in the UK. These researches indicated that genetic factor was important for VVD. We firstly exploit double-digest genotyping by sequencing (ddGBS) method to conduct GWAS based on case/control experience in this study. However, the result revealed that these SNPs were not found to significantly associate with VVD at genome wide level in broilers. As we all know, most diseases and complex traits are controlled by multiple genes, which cause these intractable traits to be controlled difficultly by the major genes or a single SNP [16]. Meanwhile, the association between a single gene and phenotypic traits is relatively weak due to the quantitative trait was controlled by multiple minor genes, which some SNP often fail to significant after Bonferroni tests in GWAS analysis. The developmental processes of skeletons are regulated by genetic factors and their interactions with environmental factors [1, 2]. Consequently, we speculated that VVD is a complex trait and is affected by a large number of small-effect variants.

The sample size is insufficient which causes the result was limited in this study. However, seven significant SNPs and four genes associated with Piglet splay leg (PSL) syndrome were obtained for the limited sample size (73 cases, 112 controls) using the linear mixed model in GWAS [17]. In a host genetic resistance to Marek’s disease study, GWAS was performed on 57 susceptible chickens (case group) and 10 resistant chickens (control group), which result in two SNPs were found to be associated with host resistance to MD [18]. In the present study, we conducted GWAS on 233 samples based on a case and control design, including 126 VVD broilers in the case group and 122 sound broilers as controls. Comprehensive consideration, we concluded that the SNPs without significant difference might be independent of the sample size.

In addition, as one of the Reduced-Representation Genome Sequencing (RRGS) methods, ddGBS was double-digest genotyping by sequencing method with high genotyping accuracy and high-density SNP markers to chickens and pigs [19]. However, there must be a great deal of Gap using ddGBS. If these sites associated with VVD are located in these Gap, which affects the sequencing results using ddGBS. Therefore, we speculated that these SNP sites associated with VVD might be affected by these gaps. In conclusion, the reason why no significant SNPs were found still needs to be further exploration. Of course, we will try to using whole genome markers to explored the leg disorder in broilers in the future study.

Hematological parameters are a valuable tool for assessing the individual’s state of health in human diagnostics. Certain economic traits are indirectly measured using serum components in animal breeding. Serum ALP, a by-product in osteoblast activity or bone formation mainly derived from liver and bone, is essential in bone mineralization and vascular calcification [6, 20]. Our previous results indicated that ALP activity was markedly inhibited and bone calcification disorders in VVD broilers [4]. Therefore, ALP is merit to the molecular mechanism of VVD and healthy leg breeding in birds. The genomic region on Chr 24 (0.22 Mb - 1.79 Mb) contains 43 SNPs associated with the ALP levels in this study. A total of 38 putative candidate protein coding genes were obtained. Among these loci and genes, we pay attention to the rs475471 (G > A) at *ST3GAL4* (ST3 beta-galactoside alpha-2, 3-sialyltransferase 4). *ST3GAL4* is associated with the pathogenesis of osteoarthritis (OA) and LDL-C level [21, 22]. In our subsequent study, we explored the relationship between *ST3GAL4* genetic variation loci and serum ALP in chicken. We determined 9 SNPs within *ST3GAL4* and *ST3GAL4* were closely related to ALP in VVD broilers (unpublished data). The findings indicated that *ST3GAL4* plays an important role in abnormal serum ALP in VVD birds (unpublished data). Furthermore, among 38 putative candidate genes, *Panx3* (Pannexin 3) was important at the early stages of bone development [23]. As a potential therapeutic target for OA, *Panx3* plays a specific catabolic role in articular cartilage [24] and can improve chondrocyte differentiation. *Panx3* merits closer attention in our follow-up study. To gain insight into the function of 38 candidate genes, GO and KEGG enrichment analyses were performed. The cartilage development, cartilage condensation GO term and candidate genes in two GO terms merit our attention in further study. According to GO enrichment analysis results, *BARX2* was focused. *BARX2*, a new homeobox gene of the Bar class, is expressed in the articular cartilage of the developing limb, suggesting that it is involved in regulation of chondrogenesis, which acts downstream of BMP signaling and in concert with Sox proteins to regulate chondrogenesis [25]. Of course, further evidence is needed to understand the specific mechanism of these candidate genes for ALP level and VVD. In pigs and chickens, the direct relationships between ALP levels and candidate genes and corresponding QTLs have not been reported at present [26]. In mice, the regions for ALP levels were located on chromosome 1 and 6 [23]. This study first time identified that a genomic region on Chr 24 (0.22 Mb - 1.79 Mb) associated with the ALP levels in Hubbard VVD birds. These results will provide some in-depth reference to the interaction relationship between VVD and ALP level.

Shank length is an important indicator for leg health in chicken. Identification of SNPs and genes affecting shank length traits were valuable for leg health in birds. For shank length, a SNP was obtained mapped on Chr 2 at position 63.02 Mb in our study. A study found that shank length was mapped to Chr 2, indicating the common effect of the chromosome on shank length and leg disease [8]. Lipid metabolism disorders was involved in leg disorder, which cause the bone swelling and deformation in VVD birds [7]. For HDL-C and serum Ca, a SNP was obtained respectively mapped on Chr 2 at position 61.93 Mb and Chr 23 at position 4.68 Mb, indicating the common effect of the Chromosome on leg disease. These results indicated that these loci and annotated genes merits closer attention.

Identification of SNP sites and candidate genes involved in VVD, growth performance and serum indicators may provide insight into disease mechanisms. For VVD, growth performance and serum indicators, several association signals and some putative genes were detected in the current study. Future study is need to confirm this finding. Our current research has provided some in-depth understanding of the interaction relationship between VVD and growth performance or serum indicators.

## Conclusion

In summary, we firstly exploited GWAS method to explore VVD trait, growth performance and serum indication in Hubbard broiler. This study excavated that some SNPs and functional candidate genes may play significant roles in the interaction relationship between ALP and VVD, which provides some reference for understanding of the pathogenesis of VVD and improving leg healthy in poultry industry.

## Methods

### Animals and sample preparation

Hubbard broilers (*n* = 56,000) were reared in Zhonghong Sanrong Group Co., Ltd.; Liaoning, China. A total of 15 individuals with leg weakness were anesthetized by an intravenous wing injection of sodium pentobarbital (0.2%, 40 mg/kg body weight) in the wing vein. Subsequently, these individuals were euthanized by intravenous KCl (1–2 mg/kg). Subsequently, these individuals were anatomical. According to clinical examination, anatomical results and the bent degree of abnormal leg, Hubbard broilers with leg weakness were diagnosed as VVD at 38 -day- old [1, 3]. Then, 126 VVD broilers (case group) and 122 sound broilers (control group) were randomly selected, respectively. The leg disorder in the phenotype broiler was visually classified as normal or affected (0 / 1, respectively). Blood samples were collected from wing vein and stored at − 20 °C until DNA isolation. A total of seven serum indicators including serum alkaline phosphatase (ALP), calcium (Ca), phosphorus (P), triglyceride (TG), total cholesterol (T-CHO), high-density lipoprotein cholesterol (HDL-C), low-density lipoprotein cholesterol (LDL-C) were measured [4]. Three growth traits including body weight, shank length, and shank girth of these birds were measured using a weighing scale and caliper [4].

### DNA isolation and sequencing

DNA was extracted from blood tissue using the Qiagen DNeasy Blood and Tissue Kit (Qiagen, Hilden, Germany) and diluted to a normal 50 ng/μL. The DNA quality satisfied the requirements for library construction of double-digest genotyping-by-sequencing (ddGBS) [19]. The quality of DNA libraries was estimated by Agilent 2100 Bioanalyzer (Agilent, Santa Clara, CA, U.S.A.) and Qubit2.0 Fluorometer (Thermo, MA, U.S.A.). Ultimately, these libraries were sequenced on the Illumina Hiseq X Ten platform with 2 × 150-bp paired-end reads were generated.

### Genotyping and quality control

Removing these reads of that were polluted by adapter sequence and the low-quality reads from raw reads. The clean reads were aligned to the chicken reference genome (Gallus_gallus-6.0) using Bowtie2 and TASSEL GBS analysis pipeline was used to call SNP [24, 27]. The quality control of these genotyping data was performed through VCFtools [28] to remove the SNPs, in accordance with these strict parameters: a minor allele frequency (MAF) > 0.05; the quality of genotypes above 98 (GQ ≥ 98%) and depth ≥ 5; consistent with Hardy-Weinberg equilibrium; only biallelic markers were retained; animals with missing rate < 0.4. According to the information of the remaining SNPs to imputed the missing genotypes using Beagle 4.0 software [19, 29]. Meanwhile, SNPs on sex chromosomes were removed. Finally, a total of 233 samples (120 case and 113 control) and 256, 599 markers were kept for further analysis after quality control.

### Genome wide association study

Principal component analysis (PCA) was conducted using GCTA software to determine whether stratification exists in our study population. All SNPs were pruned to ensure their independence using the option of “indep-pairwise 25 5 0.2” in PLINK software to avoid the highly correlated SNP may distort the resulting PCs. A total of 33,030 independent effective tests were therefore obtained. GWAS was accomplished with the single-locus mixed linear model procedure implemented in GEMMA software using effective SNPs.

$$\boldsymbol{y}=\boldsymbol{W}\boldsymbol{\alpha } +\boldsymbol{X}\boldsymbol{\beta } +\boldsymbol{g}+\boldsymbol{e}$$

where Y is the vector of phenotypes (VVD trait, serum indicator and growth trait), ***α*** is the fixed effect vector including gender (male; female), chicken house (1; 2), ***β*** is the effect size of the genotype, g is a n-vector of random individual genetic effects, e is a vector of residuals; W, X are incidence matrix corresponding to the fixed effects in ***α*** and the genotype effect of ***β***. Association analysis results were adjusted using Bonferroni correction (*P* < 0.05/ N). The significance threshold of GWAS analysis was 0.05/33030 = 1.51e-6, where 33,030 is the number of independent markers. The significance threshold of GWAS analysis was 1/33030 = 3.02e-5 at the suggestive levels. The manhattan and Q-Q plots were obtained from the GWAS results with the CMplot package within the R software.

### Post-GWAS analysis

To identify the positional candidate genes that are potentially associated with VVD trait, serum indicator and growth trait, the genomic regions that were close to the significant SNPs with a distance less than 300 Kb were selected. These regions were then referenced against the *Gallus gallus* reference genome (GRCg6a) using the Ensembl Genome Browser to find genes that are located in the vicinity of the significant SNPs [16]. The candidate genes were performed Gene Ontology (GO) and Kyoto Encyclopedia of Gene and Genomes database (KEGG) enrichment analysis with the cutoff of *P*-values < 0.05 [30–32]. In order to screen VVD susceptibility genes, the candidate genes from GWAS and candidate genes from previous RNA-seq [2] were overlapped.

## Supplementary Information

**Additional file 1: Table S1.** The phenotypic information of VVD and sound broile. **Table S2.** Annotated candidate genes to VVD trait. **Table S3.** Annotated candidate genes to ALP trait. **Table S4.** GO enrichment analysis of the candidate genes for VVD trait in Valgus-varus Deformity Broilers. **Table S5.** KEGG enrichment analysis of the candidate genes for VVD trait. **Table S6.** GO enrichment analysis of the candidate genes for ALP trait in Valgus-varus Deformity Broilers. **Table S7.** KEGG enrichment analysis of the candidate genes for ALP trait in Valgus-varus Deformity Broilers.

**Additional file 2: Figure S1.** Distribution of SNPs on each chromosome. **Figure S2.** Distribution of SNPs on chromosome position. **Figure S3.** Population structure evaluated by the first two principal components. **Figure S4.** Manhattan and quantile-quantile (QQ) plot on Serum TG, T-CHO, LDL-C and P. **Figure S5.** Manhattan and quantile-quantile (QQ) plot on body weight and shank girth.

## Data Availability

The GWAS datasets generated and/or analyzed during the present study are available at NCBI project PRJNA720768 with accession number SRR14251137–39.

## Abbreviations

VVD: Valgus-varus deformity
SNP: Single nucleotide polymorphism
ALP: Alkaline phosphatase
Ca: Calcium
P: Phosphorus
TG: Triglyceride
T-CHO: Total cholesterol
HDL-C: High-density lipoprotein cholesterol
LDL-C: Low-density lipoprotein cholesterol
BW: Body weight
SL: Shank length
SG: Shank girth
ddGBS: Double-digest genotyping by sequencing (ddGBS)
GWAS: Genome-wide association study
RRGS: Reduced-Representation Genome Sequencing
FST: F-Statistics
LD: Linkage disequilibrium
PCs: Principal components
Chr: Chromosome
BARX2: BARX homeobox 2
HYLS1: Centriolar and ciliogenesis associated
GO: Gene Ontology
KEGG: Kyoto Encyclopedia of Gene and Genomes database
ST3GAL4: ST3 beta-galactoside alpha-2,3-sialyltransferase 4
