# Supplementary material for: Genome-wide association study identifies SNPs for growth performance and serum indicators in Valgus-varus deformity broilers (Gallus gallus) using ddGBS sequencing
Source: BMC Genomics. 2022 Jan 6;23:26. doi: 10.1186/s12864-021-08236-3 (PMC8734266; doi:10.1186/s12864-021-08236-3)
Supplement: Supplementary file 2 — Additional file 2: Figure S1. Distribution of SNPs on each chromosome. Figure S2. Distribution of SNPs on chromosome position. Figure S3. Population structure evaluated by the first two principal components. Figure S4. Manhattan and quantile-quantile (QQ) plot on Serum TG, T-CHO, LDL-C and P. Figure S5. Manhattan and quantile-quantile (QQ) plot on body weight and shank girth. [file 12864_2021_8236_MOESM2_ESM.pdf]

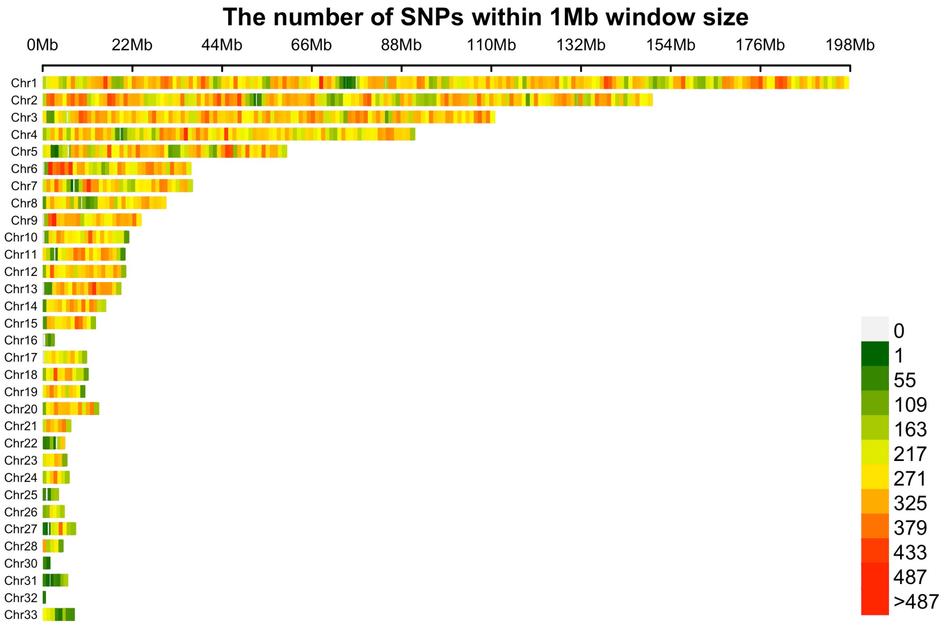

Figure S1. Distribution of SNPs on each chromosome.

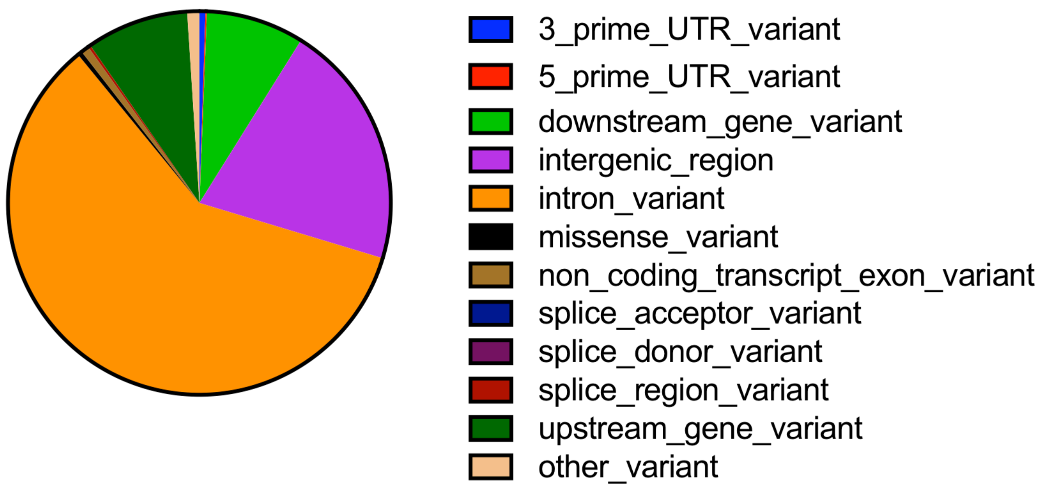

Figure S2. Distribution of SNPs on chromosome position.

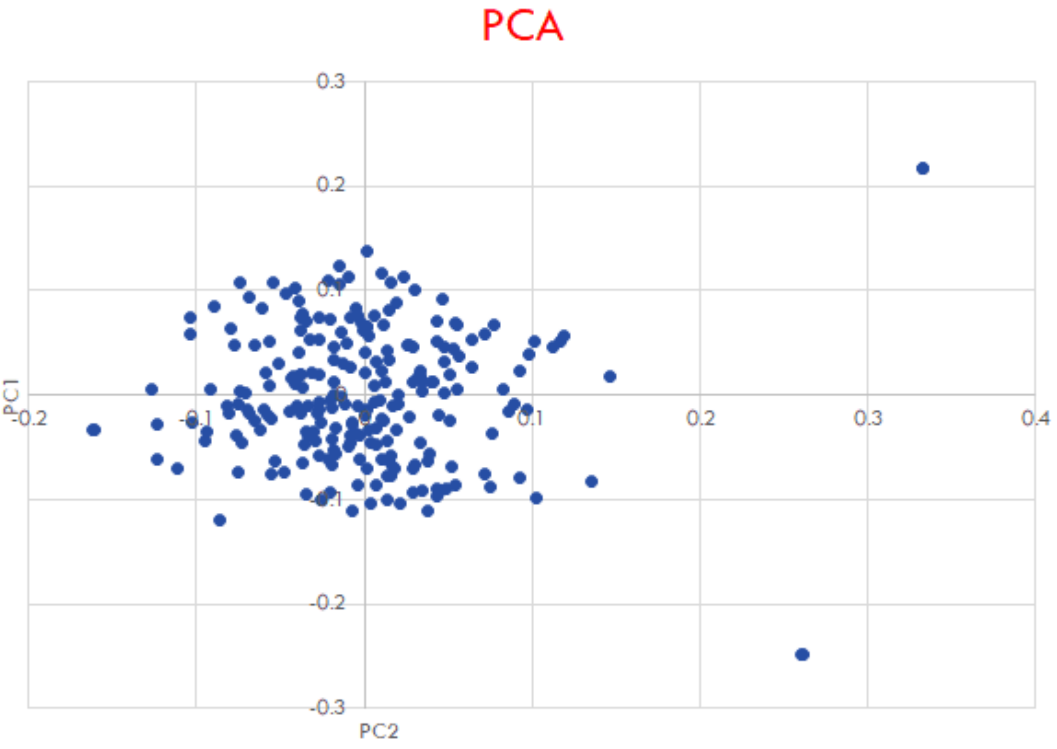

Figure S3. Population structure evaluated by the first two principal components.

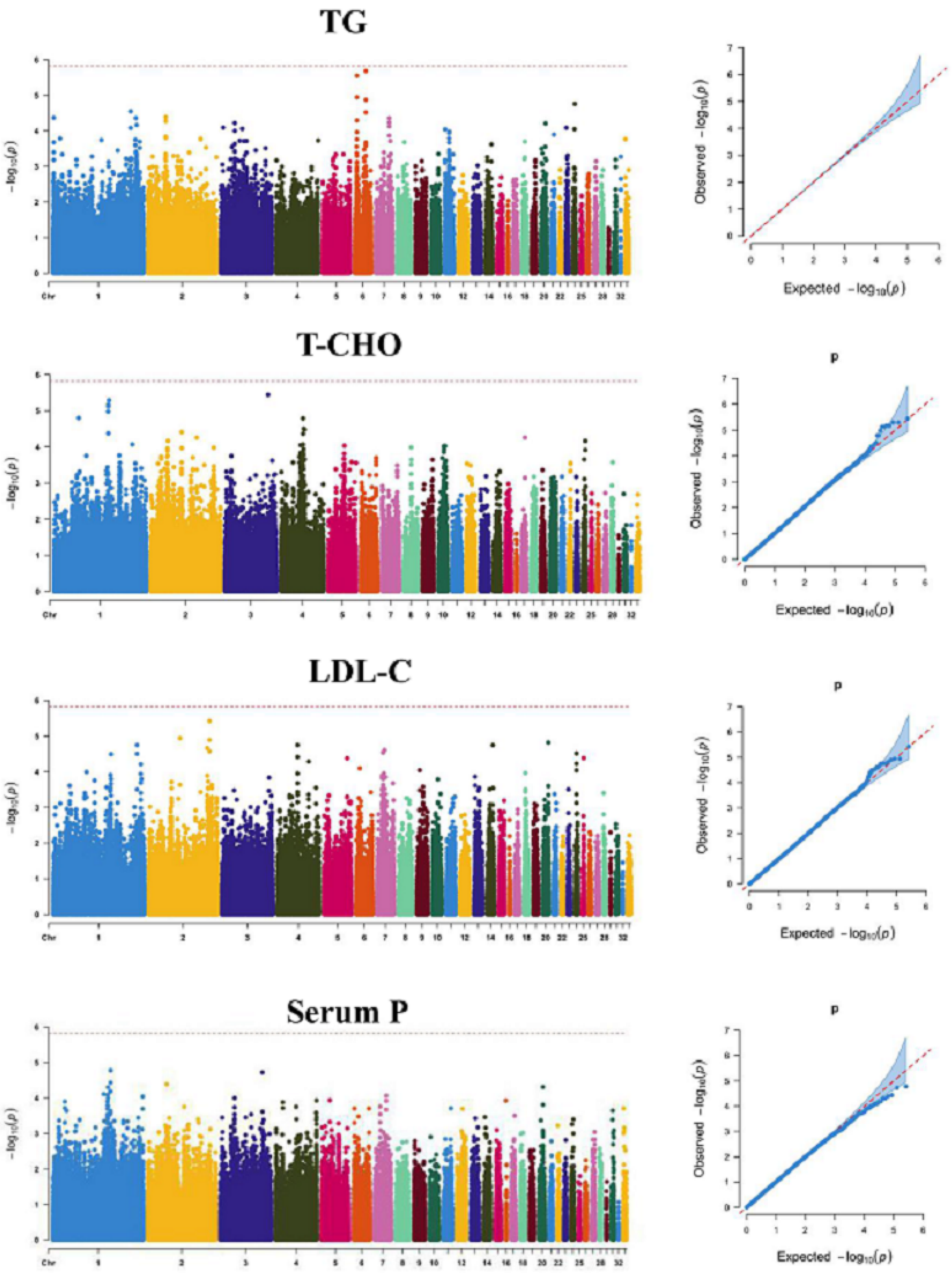

Figure S4. Manhattan and quantile-quantile (QQ) plot on Serum TG, T-CHO, LDL-C and P.

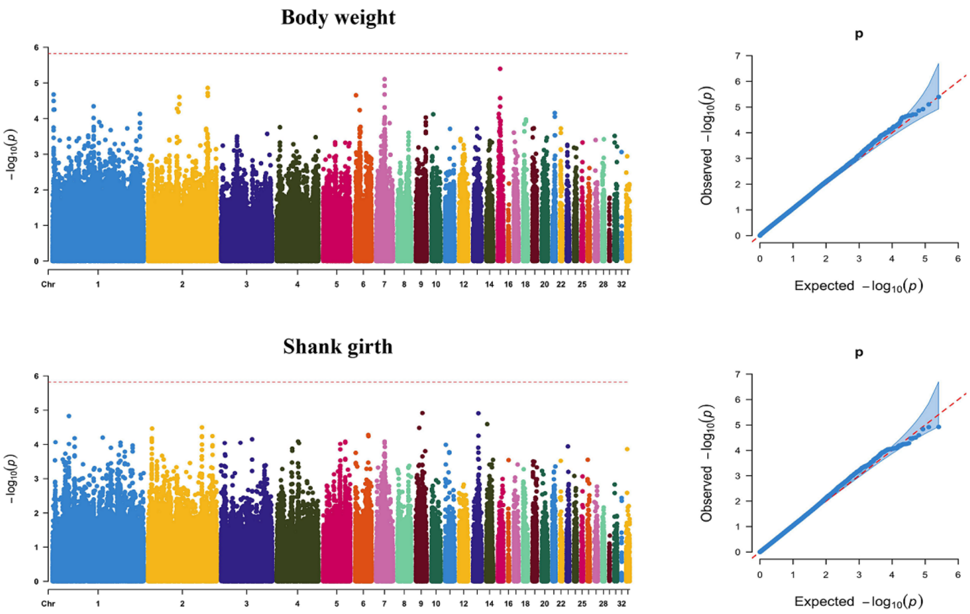

Figure S5. Manhattan and quantile-quantile (QQ) plot on body weight and shank girth.
